# IMPROVING REHABILITATION OUTCOMES FOR PEOPLE AGING WITH LONG-TERM HAND IMPAIRMENTS

**DOI:** 10.1093/geroni/igad104.0129

**Published:** 2023-12-21

**Authors:** Jon Sanford, Susan Lee, Yi-An Chen, Sutanuka Bhattacharjya, Emily Buchman, Martin Norgaard, Gil Weinberg

**Affiliations:** Dept. of Occupational Therapy, Georgia State University, Atlanta, Georgia, United States; Georgia State University, Atlanta, Georgia, United States; Dept. of Occupational Therapy, GA State, Atlanta, Georgia, United States; Georgia State University, Atlanta, Georgia, United States; Dept. of Occupational Therapy, GA State, Atlanta, Georgia, United States; Music Education, GA State, Atlanta, Georgia, United States; School of Music, GA Tech, Atlanta, Georgia, United States

## Abstract

Despite the importance of hand and upper extremity function in ADL performance and successful aging-in-place, contextual and engaging in-home rehabilitation interventions that promote use, feedback of progress, and long-term monitoring of changes in functional ability among individuals with chronic upper limb deficits are lacking. This presentation will present the findings from three TechSAge pilot projects focused on the use of technology to support in-home rehabilitation for individuals aging with long-term upper extremity disabilities. DigiHand, is a smartphone-based application combined with sensor-embedded objects used in daily activities (e.g., coffee cup) that enables users to self-manage their own rehabilitation, obtain feedback on progress to refine movements, and allow remote monitoring by therapists. Similarly, KeyStroke is an in-home piano training mobile app that provides engaging music exercises to promote long-term rehabilitation and therapists’ monitoring of progress of stroke survivors. Finally, 3DP for AT is generating design requirements to support the development of an open-source software tool that would allow assistive technology service providers, who are novice users of 3D printers, to create and fabricate highly customized 3D-printed assistive devices that are more durable, aesthetic, and will better meet the unique needs of people aging with long-term hand impairments.

